# Evaluation of proximal-distal velocity gradient in spastic middle cerebral artery after aneurismal subarachnoid hemorrhage

**DOI:** 10.1186/cc12277

**Published:** 2013-03-19

**Authors:** M Gotti, F Stretti, S Pifferi, V Conte, M Zara, N Stocchetti

**Affiliations:** 1 Fondazione IRCCS Ca' Granda-Ospedale Maggiore Policlinico, Milan, Italy; 2 Fondazione IRCCS Ca' Granda-Ospedale Maggiore Policlinico, Milan University, Milan, Italy

## Introduction

Delayed cerebral ischemia (DCI) worsens neurological outcome in patients with aneurysmal subarachnoid hemorrhage (aSAH). DCI pathogenesis is multifactorial and not completely understood, but vasospasm plays a central role. Transcranial color-coded duplex sonography (TCCDS) is a non-invasive bedside tool to explore cerebral vessels, but specificity is still suboptimal. The aim of this study was to evaluate the proximal-distal gradient of mean cerebral blood flow velocity (CBF-V) in middle cerebral artery (MCA) as a possible indicator of critical vasospasm.

## Methods

Consecutive aSAH patients (WFNS 1 to 5, age 18 to 80 years) admitted to NeuroICU between November 2011 and September 2012 were included in this study. TCCDS was used to assess CBF-V in MCA of both sides: we defined TCCDS vasospasm as CBF-V >120 cm/second. Magnetic resonance angiography (MRA, 3D TOF HR) was performed to evaluate vasospasm at early (<3 days) and delayed (7 to 10 days) time points. When patients underwent MRA, in the same clinical conditions, we recorded CBF-V with TCCDS at two levels: at the origin (proximal CBF-V) and at the end (distal CBF-V) of the MCA. Then we calculated the absolute value of proximal-distal CBF-V gradient for MCA of both sides. We defined clinical vasospasm as the appearance of a new neurological deficit confirmed by imaging. The relationships between the absolute value of TCCDS CBF-V gradient and (1) TCCDS vasospasm, (2) MRA vasospasm and (3) clinical vasospasm were explored.

## Results

We included 26 consecutive aSAH patients (WFNS 1 to 5, age 57 ± 12 years). The absolute value of MCA CBF-V gradient was higher in: (1) vessels affected by TCCDS vasospasm when compared with vessels not affected (63 cm/second, IR 23 to 85 and 15 cm/second IR 7 to 23 respectively, *P <*0.001); (2) vessels affected by MRA vasospasm when compared with vessels not affected (43 cm/second, IR 18 to 78 and 13 cm/second IR 6 to 22, *P <*0.001); and (3) in patients who developed clinical vasospasm than in patients who did not (56 cm/second IR 11 to 124 and 16 cm/second IR 8 to 30 respectively, *P <*0.01). Considering only the subset of MCAs affected by MRA vasospasm, the CBF-V gradient was higher when clinical vasospasm was also present (75 cm/ second IR 56 to 124 and 23 cm/second IR 17 to 30 respectively, *P <*0.05).

## Conclusion

Proximal-distal CBF-V gradient of MCA might be a reliable indicator of critical vasospasm but further studies are warranted to define threshold values and specificity in aSAH patients.

